# Assessment of asymmetric dimethylarginine and homocysteine in epileptic children receiving antiepileptic drugs

**DOI:** 10.1038/s41390-022-02132-6

**Published:** 2022-06-10

**Authors:** Asmaa A. Mahmoud, Hesham M. Aboelghar, Sabry Moawad Abdelmageed, Heba M. Abdallah, Mohamed I. Garib, Nahla M. S. Abd El Hady

**Affiliations:** 1 Department of Pediatrics, Faculty of Medicine, Menoufia University, Shebin Elkom, Egypt; 2 Department of Clinical Biochemistry and Molecular Diagnostics, National Liver Institute, Menoufia University, Shebin Elkom, Egypt; 3 Department of Clinical Pathology, National Liver Institute, Menoufia University, Shebin Elkom, Egypt

## Abstract

**Background:**

Epilepsy is a neurological disease that requires long-term antiepileptic drugs (AEDs). The old generation of AEDs may affect serum homocysteine and asymmetric dimethylarginine (ADMA) and disturb lipid levels. The aim of the study was to evaluate serum ADMA, homocysteine, lipid profile, and carotid intima-media thickness (CIMT) in epileptic children.

**Methods:**

This study was implemented on 159 epileptic children who were subdivided into 3 subgroups, with 53 receiving sodium valproate, 53 receiving levetiracetam, and 53 receiving polytherapy, for over 6 months and 53 healthy children.

**Results:**

Low-density lipoprotein, triglycerides, and cholesterol levels were increased in epileptic children (*p* < 0.001), which were higher in those receiving multidrug followed by a valproate receiver. While high-density lipoprotein was lower in those receiving multidrug more than those receiving valproate. ADMA and homocysteine levels increased in epileptic patients than in controls (*p* < 0.001). Higher ADMA was also observed in the multidrug receiver (5.78 ± 0.62), followed by the levetiracetam group (5.56 ± 0.61). Homocysteine levels were significantly higher in multidrug and valproate-treated children than those treated with levetiracetam. CIMT was significantly higher in multidrug and valproate-treated patients (*p* < 0.001).

**Conclusions:**

Long-term use of AEDs, especially old-generation polytherapy, can elevate lipid profiles, homocysteine, ADMA levels, and carotid intima-media thickness compared to the minimal effect of new AEDs.

**Impact:**

- The long-term use of antiepileptic drugs, especially old-generation polytherapy, can increase lipid profiles, homocysteine levels, ADMA, and carotid intima thickness compared to the minimal effect of new antiepileptic generation.
- A routine follow-up of these markers and a lifestyle modification are recommended to avoid cerebrovascular events as much as possible.

## Introduction

Epilepsy is considered one of the most common neurological diseases in children that require long-term therapy. Antiepileptic medication-receiving patients demonstrate several vascular risk factors, including an altered lipid profile, increased oxidative stress, and increased serum homocysteine. The ideal drug selection for each patient was based on the spectrum of activity, dose-related serious side effects, drug interactions, and costs.^1^ The atherosclerotic process can be accelerated by one phenomenal precursor, which is endothelial dysfunction. Antiepileptic drugs (AEDs) relate to high levels of homocysteine, a highly independent risk factor for asymmetric dimethylarginine (ADMA), atherosclerosis, lipoprotein (A), and impaired lipid profiles.^2^

Hyperhomocysteinemia is associated with increased ADMA concentration, which is an endogenous inhibitor of nitric oxide synthase and synthesized by arginine methylation. It is thought that both ADMA and homocysteine have adverse vascular effects because they interfere with endothelial, nitric oxide-dependent functions.^3^ ADMA levels also exacerbate oxidative stress and monocyte adhesion that correlates with the carotid media intima thickness (MIT) complex.^4^

Over 15% of children receiving AEDs are at risk of hyperhomocysteinemia. This risk may increase by using polytherapy (a combination of 2 AEDs such as carbamazepine (CBZ), valproic acid (VP), phenytoin (PHT), vigabatrin, oxcarbazepine (OXC), topiramate (TPM), lamotrigine (LTG), and clobazam).^5^ The available data on the potential effects of new-generation AEDs on hyperhomocysteinemia metabolism, including LTG, OXC, TPM, and levetiracetam (LEV), is relatively small. These drugs may be safer than their counterparts of older generations in children with epilepsy who are predisposed to developing atherosclerosis early in life, such as those with hereditary diseases of hyperhomocysteinemia metabolism or familial hyperlipidemia.^6^

### Aim

The aim of this study was an evaluation of serum ADMA, homocysteine, lipid profiles, and their correlation to CIMT in epileptic children receiving valproic acid, LEV, and polytherapy treatment.

## Subjects and methods

### Design

This case–control study nested in cross-sectional study was carried out on 159 epileptic children who were subdivided into 3 subgroups: (1) 53 receiving sodium valproate, (2) 53 receiving LEV, and (3) 53 receiving polytherapy (two or more of valproate, CBZ, topiramate, PHT, phenobarbital, and lamotrigine) for over 6 months. There were 100 boys and 59 girls with an age range of 11 months to 8 years and 50 healthy children. The age, sex, and socioeconomic status of this group matched the control group. The study period was from the first of March 2021 to the first of October 2021. They were recruited from the pediatric neurology outpatient clinic, Menoufia University Hospital. After obtaining informed written consent, the Ethics Committee of Menoufia University’s Faculty of Medicine approved this study (ID-8/3/2021.PEDI). This study details the medical histories, physical examinations, and the following investigations performed on each patient: ADMA (μmol/l), homocysteine (μmol/l), lipid profiles (mg/dl) by enzyme-linked immunosorbent assay (ELISA), and carotid intima-media thickness (CIMT).

### Diagnostic inclusion and exclusion criteria

#### Inclusion criteria

Inclusion criteria include idiopathic epileptic children receiving sodium valproate, LEV, and polytherapy treatment for at least 6 months. Healthy control has no neurological, vascular, or metabolic diseases.

#### Exclusion criteria

The exclusion criteria include the following: secondary epilepsy and a short duration of AED <6 months, obesity and hepatic diseases, any vascular disease that may affect the elasticity or thickness of the vessels (e.g., diabetes mellitus, hypertension, and sickle cell disease), and any metabolic disease that may affect lipid profiles.

### Sample collection and assay

#### Sampling

A 4 ml venous blood sample was drawn from each participant under complete aseptic conditions, allowed to clot, and then centrifuged for 15 min at 3000 rpm to separate the serum for assessing the biochemical tests (homocysteine, lipid profile, and serum human ADMA.

#### Methods

The serum homocysteine was measured using (The ARCHITECT i1000SR immunoassay analyzer). The lipid profiles [total cholesterol, triglycerides, high-density lipoprotein cholesterol (HDL-C), and low-density lipoprotein cholesterol (LDL-C)] were measured by the Beckman Coulter (Synchron CX 9 ALX) clinical Auto analyzer (Beckman Instruments, Fullerton, CA). ELISA was used to determine human ADMA by using kits provided by the Shanghai Sun Bright Biological Technology Co., Ltd. Catalog No. 201-12-1888. Human ADMA was added to monoclonal antibodies enzyme wells that were pre-coated with human ADMA monoclonal antibodies, followed by incubation. Human ADMA antibodies were then labeled with biotin and combined with streptavidin–horseradish peroxidase to form an immune complex. Finally, incubation and washing were repeated to remove the uncombined enzyme. A and B chromogen solutions were then added. Due to the acid’s impact, the liquid becomes blue and eventually yellow. The sample’s chroma was favorably associated with the concentration of the human substance human ADMA. The intra-assay and inter-assay coefficients variations were 10 and 12%, corresponding to the manufacturer’s quoted values.

### Technique description of CIMT (mm)

The model used was Duplex ultrasound on carotid artery by ESAOTE model prestige with transducer 7.5 MHz, Italy.

CIMT measures were obtained when the patient was laying supine with the neck rotated to the other side of the examination. Using three different views of each vessel, common carotid artery images were obtained to determine IMT.^7^ There were at least three IMT points measured at each vessel’s far and near walls in the thickest part of the vessel. Two longitudinal views of the sternocleidomastoid muscle were used to scan the vessel: posterolateral and anterolateral.^8^

### Sample size calculation

The sample size relied on a 95% confidence interval with 80% power, using a one-way analysis of variance (ANOVA) (with equal-size groups) and assuming an (two-sided) *α* of 0.05. Based on a previous study (ref. ^9^), the smallest mean of ADMA (µM/L) was 1.27 while the largest mean was 2.10 and SD was 1.38. The number of participants was 53 for each group of epileptic cases.

### Statistical analysis

IBM SPSS version 20 was used to analyze the data (SPSS Inc., Chicago, IL). To examine the relation between qualitative variables, a chi-square test was applied. For the quantitative data, a comparison between three groups was made using either ANOVA or Kruskal–Wallis test (non-parametric test) as appropriate. For correlation between numerical variables, Pearson’s correlation coefficient was used. A *P* value <0.05 was considered significant.

## Results

There were no significant differences for age, sex, hemoglobin, white blood cells, and platelets between patients and control groups as shown in Table 1.

**Table 1:** Demographic characteristics and CBC among the studied groups.

Characteristics of groups	Mean ± SD	Median (range)	Test of significance and *P* value	Post hoc *P* value
Age (year)				P1 = 0.07
Valproate-treated patients	3.4 ± 2.6	2 (0.92–8)	Kruskal–Wallis test = 5.45	P2 = 0.33
Levatiracetam-treated patients	4.2 ± 2.1	5 (1–8)	*P* value = 0.14	P3 = 0.15
Multidrug-treated patients	3.4 ± 1.6	4 (1–6)		P4 = 0.05
Controls	3.9 ± 2.3	3 (1–8)		P5 = 0.83
				P6 = 0.63
Sex	Male	Female		P1 = 0.16
Valproate-treated patients	36 (67.9%)	17 (32.1%)	*χ*^2^ test = 2.35	P2 = 0.84
Levatiracetam-treated patients	29 (54.7%)	24 (45.3%)	*P* value = 0.50	P3 = 0.68
Multidrug-treated patients	35 (66.0%)	18 (34.0%)		P4 = 0.23
Controls	34 (64.2%)	19 (35.8%)		P5 = 0.32
				P6 = 0.84
Hemoglobin (g/dl)				P1 = 0.95
Valproate-treated patients	11.9 ± 0.78	12 (10–12.8)	ANOVA test = 1.15	P2 = 0.11
Levatiracetam-treated patients	11.9 ± 1.3	11.8 (10.2–16)	*P* value = 0.33	P3 = 0.45
Multidrug-treated patients	11.6 ± 0.65	11.5 (10.8–12.6)		P4 = 0.12
Controls	11.8 ± 0.47	11.8 (11–13)		P5 = 0.49
				P6 = 0.39
White blood cells (×10^3^/mm^3^)				P1 < 0.001**
Valproate-treated patients	6.5 ± 1.2	6.9 (5–8.1)	ANOVA test = 11.41	P2 = 0.02*
Levatiracetam-treated patients	8.2 ± 1.9	7.8 (5.7–11.5)	*P* value < 0.001**	P3 < 0.001**
Multidrug-treated patients	7.3 ± 1.8	6.5 (5–10.5)		P4 = 0.006*
Controls	8.1 ± 1.9	8.5 (5–11.5)		P5 = 0.87
				P6 = 0.01*
Platelet (×10^3^/mm^3^)				P1 = 0.30
Valproate-treated patients	263.4 ± 41.0	250 (213–355)	ANOVA test = 1.20	P2 = 0.69
Levatiracetam-treated patients	273.9 ± 60.9	269 (198–452)	*P* value = 0.31	P3 = 0.24
Multidrug-treated patients	259.3 ± 42.3	260 (187–319)		P4 = 0.15
Controls	275.2 ± 59.9	267 (194–452)		P5 = 0.89
				P6 = 0.12

P1: valproate-treated patients versus levatiracetam-treated patients; P2: valproate-treated patients versus multidrug-treated patients; P3: valproate-treated patients versus controls. P4: levatiracetam-treated patients versus multidrug-treated patients; P5: levatiracetam-treated patients versus controls; P6: multidrug-treated patients versus controls.

*Significant difference. **Highly significant difference.

As for lipid profiles, LDL, triglycerides, and cholesterol levels were significantly higher in epileptic children than the control groups (*P* < 0.001), higher in those receiving multidrug followed by valproate receiver while the LEV group was less affected. In contrast, HDL was lower in those receiving multidrug more than those receiving valproate with a significant difference compared with controls, as shown in Table 2.

**Table 2:** Comparison of lipid profile among the studied groups.

Lipid profile	Mean ± SD	ANOVA test and *P* value	Post hoc *P* value
LDL (mg/dl)			P1 < 0.001**
Valproate-treated patients	139.7 ± 14.6	Test = 294.19	P2 < 0.001**
Levatiracetam-treated patients	123.1 ± 8.5	*P* value < 0.001**	P3 < 0.001**
Multidrug-treated patients	151.3 ± 14.1		P4 < 0.001**
Controls	83.0 ± 12.5		P5 < 0.001**
			P6 < 0.001**
HDL (mg/dl)			P1 < 0.001**
Valproate-treated patients	30.1 ± 1.7	Test = 154.85	P2 = 0.30
Levatiracetam-treated patients	35.3 ± 1.8	*P* value < 0.001**	P3 < 0.001**
Multidrug-treated patients	30.5 ± 1.8		P4 < 0.001**
Controls	36.7 ± 2.4		P5 < 0.001**
			P6 < 0.001**
Cholesterol (mg/dl)			P1 < 0.001**
Valproate-treated patients	179.9 ± 16.5	Test = 212.95	P2 < 0.001**
Levatiracetam-treated patients	149.4 ± 23.6	*P* value < 0.001**	P3 < 0.001**
Multidrug-treated patients	193.5 ± 16.9		P4 < 0.001**
Controls	109.6 ± 16.1		P5 < 0.001**
			P6 < 0.001**
Triglyceride (mg/dl)			P1 < 0.001**
Valproate-treated patients	170.2 ± 10.8	Test = 354.40	P2 < 0.001**
Levatiracetam-treated patients	148.7 ± 9.6	*P* value < 0.001**	P3 < 0.001**
Multidrug-treated patients	244.6 ± 38.4		P4 < 0.001**
Controls	110.3 ± 14.9		P5 < 0.001**
			P6 < 0.001**

P1: valproate-treated patients versus levatiracetam-treated patients; P2: valproate-treated patients versus multidrug-treated patients; P3: valproate-treated patients versus controls. P4: levatiracetam-treated patients versus multidrug-treated patients; P5: levatiracetam-treated patients versus controls; P6: multidrug-treated patients versus controls.

**Highly significant difference.

The analysis of ADMA and homocysteine levels show a significant increase in both markers in patients compared to controls (*P* < 0.001). But within the higher ADMA mean in the multidrug receiver (5.78 ± 0.62) followed by the LEV group (5.56 ± 0.61), the homocysteine levels were significantly higher in multidrug- and valproate-treated children compared to LEV ones. CIMT was also significantly higher in multidrug- and valproate-treated patients compared to the control group (*P* < 0.001) with no difference in the LEV group (Table 3).

**Table 3:** Asymmetric dimethylarginine (ADMA), homocysteine and carotid intima media thickness (CIMT) among the studied groups.

Biomarkers	Mean ± SD	ANOVA test and *P* value	Post hoc *P* value
ADMA (μmol/l)			P1 < 0.001**
Valproate-treated patients	4.40 ± 0.75	Test = 630.41	P2 < 0.001**
Levatiracetam-treated patients	5.56 ± 0.61	*P* value < 0.001**	P3 < 0.001**
Multidrug-treated patients	5.78 ± 0.62		P4 = 0.06
Controls	1.27 ± 0.35		P5 < 0.001**
			P6 < 0.001**
Homocysteine (μmol/l)			P1 = 0.28
Valproate-treated patients	9.2 ± 1.7	Test = 32.01	P2 = 0.002*
Levatiracetam-treated patients	8.7 ± 2.1	*P* value < 0.001**	P3 < 0.001**
Multidrug-treated patients	10.5 ± 3.3		P4 < 0.001**
Controls	6.4 ± 1.2		P5 < 0.001**
			P6 < 0.001**
CIMT (mm)			P1 < 0.001**
Valproate-treated patients	0.33 ± 0.02	Test = 42.88	P2 < 0.001**
Levatiracetam-treated patients	0.32 ± 0.01	*P* value < 0.001**	P3 < 0.001**
Multidrug-treated patients	0.35 ± 0.02		P4 < 0.001**
Controls	0.31 ± 0.02		P5 = 0.09
			P6 < 0.001**

P1: valproate-treated patients versus levatiracetam-treated patients; P2: valproate-treated patients versus multidrug-treated patients; P3: valproate-treated patients versus controls; P4: levatiracetam-treated patients versus multidrug-treated patients; P5: levatiracetam-treated patients versus controls; P6: multidrug-treated patients versus controls.

**Highly significant difference.

**Fig. 1:**
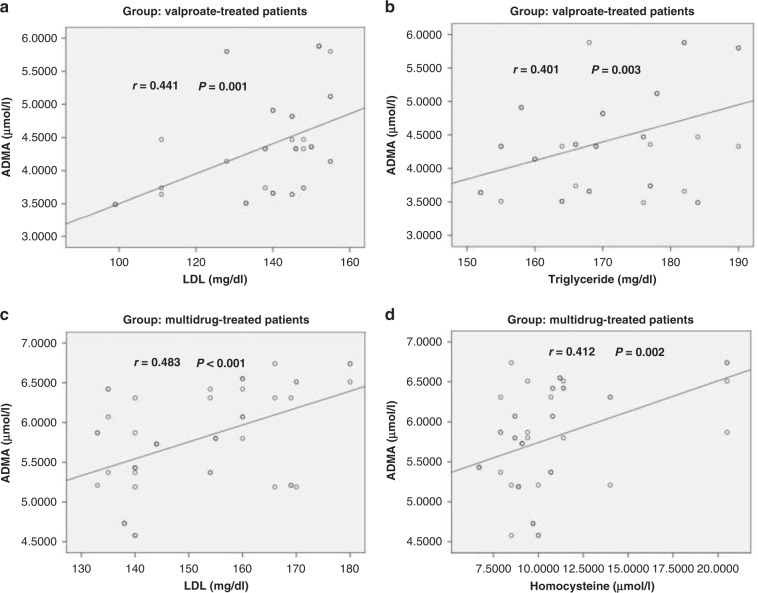
Correlation between asymmetric dimethylarginine (ADMA) and other parameters among each group. **a** Correlation between ADMA and low denisty lipoprotein (LDL) in valproate-treated patients. **b** Correlation between ADMA and triglycerides in valproate-treated patients. **c** Correlation between ADMA and low denisty lipoprotein (LDL) in multidrug-treated patients. **d** Correlation between ADMA and homocysteine in multidrug-treated patients.

There was a significant positive correlation between serum ADMA and triglycerides in the valproate group and with homocysteine in the multidrug receiver, while there was a highly significant positive correlation between ADMA and HDL in both groups. On the other hand, there was no significant correlation between ADMA and the parameters in the LEV group. There was a significant positive correlation between ADMA and homocysteine (Table 4, and Fig. 1).

**Table 4:** Correlation between asymmetric dimethylarginine (ADMA) and other parameters among each group.

Group	Parameters	ADMA (μmol/l)	
		*r*	*P* value
Valproate-treated patients	LDL (mg/dl)	0.441	0.001**
	HDL (mg/dl)	−0.076	0.59
	Cholesterol (mg/dl)	−0.091	0.52
	Triglyceride (mg/dl)	0.401	0.003*
	CIMT (mm)	−0.157	0.26
	Homocysteine (μmol/l)	−0.152	0.28
Levatiracetam-treated patients	LDL (mg/dl)	−0.234	0.09
	HDL (mg/dl)	0.217	0.12
	Cholesterol (mg/dl)	−0.242	0.08
	Triglyceride (mg/dl)	−0.126	0.37
	CIMT (mm)	0.039	0.78
	Homocysteine (μmol/l)	0.007	0.96
Multidrug-treated patients	LDL (mg/dl)	0.483	<0.001**
	HDL (mg/dl)	−0.059	0.67
	Cholesterol (mg/dl)	−0.218	0.12
	Triglyceride (mg/dl)	0.098	0.48
	CIMT (mm)	0.204	0.14
	Homocysteine (μmol/l)	0.412	0.002*

*Significant difference. **Highly significant difference.

## Discussion

To our knowledge, this is one of the most important studies to evaluate the risk of atherosclerosis in children who received old and new AEDs associated with hyperhomocysteinemia and elevated ADMA levels in our center.

As for the lipid profile (triglyceride, LDL, cholesterol, HDL) study, there was a significant elevation of lipid profiles in patients compared with the controls groups, regardless of the type of AEDs received. Comparing patients who received the old drug (group A) with group B showed a significant elevation of these markers in group A, while those receiving polytherapy had higher levels compared to both groups A and B, except for HDL that was not significantly affected in the LEV group. Some serum lipids, such as total cholesterol (TC) and LDL-C, promote atherosclerosis, while others act as a strong defense against it (e.g., HDL-C). The ratio between the cholesterol fractions (TC/HDL and LDL/HDL) is a better indicator for developing atherosclerosis in patients receiving long-term anticonvulsants.^10^

The antiepileptic medication effects on the lipid profiles were controversial in previous studies. Some of these studies illustrated an elevation of triglycerides and HDL levels.^11–13^ Also, there was an elevation of LDL values.^14,15^ But Eiris et al.^11^ reported a decrease in LDL levels in patients treated with AEDs.

The possible explanation for decreased serum lipids with valproate is valproate’s enzyme inhibitory effect. Glucuronidation is the major route of valproate biotransformation. Valproate or its metabolites may inhibit the glucuronidase enzyme, resulting in reduced triglyceride, LDL, and HDL production.^11^ Horie and Suga^16^ also observed that valproate treatment increased hepatic peroxisomal oxidation, reducing LDL-C and apolipoprotein B. Valproate-induced weight gain may result in insulin resistance, resulting in dyslipidemia and hyperinsulinemia.^17^

Akosy et al.^18^ observed that the serum lipid profile and thyroid function tests did not affect long-term LEV administration. As a result, it appears that LEV outperforms valproate benefits. However, Kim et al.^19^ showed a significant increase in LDL-C levels in LEV-treated patients. But no effect of LEV was seen on vitamin B12, triglyceride, total cholesterol, or HDL-C levels. There was a highly significant increase in homocysteine levels in patients compared to those in the control group. These higher levels were reported in the multidrug group followed by the valproic acid group.^20^

The mechanism of valproate that induces hyperhomocysteinemia is not fully understood. Moreover, the results of the possible effects of new-generation AEDs, such as OXC, lamotrigine, topiramate, and LEV, on the metabolism of homocysteine are limited.^6^ Belcastro et al.^21^ demonstrated that newer AEDs such as TPM and OXC may lead to hyperhomocysteinemia, while AEDs as LEV and LTG had no effect on homocysteine level.

The relationship between the use of new AEDs and homocysteine levels was studied in Korean patients with newly diagnosed epilepsy and treated with OXC, LEV, or TPM as monotherapy. Kim et al.^19^ observed a statistically significant elevation in homocysteine concentration throughout each drug’s therapy, but these changes are within the physiological concentration range.

Gorgone et al.^22^ reported that 30% of patients with brain atrophy were associated with the use of different AEDs and an elevated homocysteine concentration. So there was a correlation between homocysteine-induced neuronal injury, oxidative stress, and excitotoxicity.

In another research by Ono et al.^23^, they found an increased risk of hyperhomocysteinemia in patients taking prolonged, multiple AED therapies over 7 years (>7 years). Also, Vilaseca et al.^24^ found elevated total homocysteine levels in children with epilepsy using AEDs for an extended period. These prospective studies were conducted to assess the time needed until reaching the hyperhomocysteinemia cutoff value for using certain AEDs that induce hyperhomocysteinemia. So, long-term morbidity associated with AEDs can be prevented by switching these drugs before reaching the critical cutoff value for hyperhomocysteinemia.

Perhaps AED–gene interactions have a role in the development of hyperhomocysteinemia; patients getting CBZ or PHT have higher homocysteine levels if they were homozygous TT genotype, while those who taking valproic acid had lower levels.^25^ In contrast, Vurucu et al.^26^ did not confirm the correlation between the genotypes of 677T variants of hyperhomocysteinemia and the methylenetetrahydrofolate reductase (MTFR) gene polymorphism in patients with epilepsy and treated with valproate monotherapy and CBZ.

ADMA levels in the patient groups were significantly higher than the control group but remained within normal levels (<15 μmol/l), with higher levels in the multidrug group than the other groups and a significant positive correlation with homocysteine. Khanna et al.^9^ found an elevation of both ADMA and homocysteine levels in children receiving valproate and OXC after 6 months of therapy. Similarly, Oz et al.^27^ illustrated the effect of valproate and OXC therapy on the ADMA levels in the Indian population. Hyperhomocysteinemia leads to increased ADMA production, which lowers the nitric oxide levels increasing the risk of atherosclerosis.^28,29^ A significant elevation of ADMA, homocysteine and triglyceride levels was reported in children receiving valproate without elevation in LDL levels.^2^ Sniezawska et al.^30^ observed a significant link between ADMA levels and hyperhomocysteine in children with epilepsy. Yet another study reported a significant increase in ADMA and homocysteine levels in children on OXC therapy with no significant correlation between ADMA and homocysteine.^31^

A recent study reported no significant difference as regard lipid profile and homocysteine levels between epileptic children and controls with a significant positive correlation between homocysteine levels and TG levels in boys without epilepsy.^32^

CIMT was significantly higher in patients receiving multidrug and valproate compared to the control group, with no difference in the LEV group. Ksoo et al.^33^ illustrated a significant increase in CIMT values in children receiving PHT and CBZ after 3 months of therapy. Recent studies showed that patients with epilepsy who received AEDs might exhibit an increased risk of myocardial infarction, stroke, and cardiovascular death that may be triggered by affecting ADMA and homocysteine concentration and serum lipid levels.^34–36^

### Limitations of the study

Because patients were not screened genetically for the CBS genes and MTHFR, both known to have a role in homocysteine metabolism, we recommend the genetic workup of homocysteine and ADMA for early detection of the risk factors of vascular disease in children receiving AEDs for a long duration.

## Conclusion

Long-term use of AEDs, especially old-generation polytherapy, have risks of elevated lipid profiles, homocysteine levels, ADMA, and increased carotid intima thickness compared to the minimal effect of new antiepileptic generation. Routine follow-up of these markers is recommended with lifestyle modification to avoid cerebrovascular events as much as possible.

## Supplementary information

Supplementary file.

## Data Availability

The datasets generated during and/or analyzed during the current study are available from the corresponding author on reasonable request.
