# Supplementary material for: Assessment of asymmetric dimethylarginine and homocysteine in epileptic children receiving antiepileptic drugs
Source: Pediatr Res. 2022 Jun 10;92(6):1606–12. doi: 10.1038/s41390-022-02132-6 (PMC9771805; doi:10.1038/s41390-022-02132-6)
Supplement: Supplementary file 1 — Supplementary file. [file 41390_2022_2132_MOESM1_ESM.pdf]

Assessment of Asymmetric Dimethyla~~ A~~rginine~~,~~ and Homocysteine in Epileptic Children R~~r~~eceiving Antiepileptic Drugs

Short running title: Epileptic children receiving old and new antiepileptic drugs.

Disclosure: The authors declare no financial interest or any other conflicts of interest related to this work.

Keywords~~Key words~~: Asymmetric Dimethyla~~ A~~rginine (ADMA); Homocysteine; Lipid Profile; Epilepsy.

Key Message:

* The long-term~~Long term~~ use of antiepileptic drugs, especially old generation polytherapy, can increase~~have risks of elevation of~~ lipid~~s~~ profiles, ~~homocystien~~homocysteine levels, ADMA, and ~~increase~~ carotid intima thickness compared to the minimal effect of new antiepileptic generation.

*A r~~R~~outine follow-up~~follow up~~ of these markers and a lifestyle~~ is recommended with life style~~ modification are recommended to avoid cerebrovascular events as much as possible.

Abstract

Background: Epilepsy is a neurological disease that requires long-term antiepileptic drugs (AEDs). The old generation of AEDs may affect serum homocysteine~~,~~ and asymmetric dimethylarginine~~dimethyl arginine~~ (ADMA)~~,~~ and ~~disturblipid~~disturb lipid ~~levels.**Aim**~~levels.

Aim: ~~Evaluationofserum~~To evaluate serum ADMA, homocysteine, lipid profile, and carotid intima-media~~intima media~~ thickness (CIMT) in epileptic children.

~~**Methods:**This~~**Methods:** This study was implemented on 159 ~~epilepticchildren~~epileptic children who were subdivided into 3~~3~~ subgroups, with 53 receiving sodium valproate, 53 receiving levetiracetam, and 53 receiving polytherapy, ~~respectively~~ for over~~more than~~ 6 months.

Results: Low-density~~Low density~~ lipoprotein, triglycerides, and cholesterol levels were increased ~~inepileptic~~in epileptic children (p<0.001), which were~~being~~ higher in those receiving multidrug followed by a valproate receiver~~receiver~~. While high-density lipoprotein was lower in those receiving multidrug more than those receiving valproate,~~.~~ ~~Both~~ ADMA and homocysteine levels ~~were~~ increased in epileptic patients (P<0.001). Higher ADMA was also observed in the multidrug receiver (5.78±0.62), followed by the levetiracetam group (5.56±0.61). Homocysteine levels were significantly higher in multidrug and valproate-treated~~valproate treated~~ children than~~compared to~~ those treated with levetiracetam~~ ones~~. ~~CIMTwas~~CIMT was significantly higher in multidrug and valproate-treated~~valproate treated~~ patients (p<0.001).

Conclusion: Long-term~~Long term~~ use of AEDs, especially old generation polytherapy, can elevate~~have risks of elevated~~ lipid profiles, ~~homocystien~~homocysteine, ADMA levels, and ~~increased~~ carotid intima thickness compared to the minimal effect of new AEDs.

Introduction:

Epilepsy is considered one of the most common ~~neurologicaldiseases~~neurological diseases in children that require long-term~~long term~~ ~~therapy.Antiepileptic~~therapy. Antiepileptic medication receiving patients demonstrate several~~demonstrate a number of~~ vascular risk factors, including an altered lipid profile, increased oxidative stress, and increased~~an increase in~~ serum homocysteine. The i~~I~~deal drug selection for each patient was based on the spectrum of activity, dose-related serious side effects, drug interactions, and costs.[1]~~.~~ The atherosclerotic~~Atherosclerotic~~ process can be accelerated by one phenomenal precursor, which is endothelial dysfunction. Antiepileptic drugs (AEDs) relate ~~have a relationship~~ to high levels ~~ofhomocysteine~~of homocysteine, a highly independent risk factor for asymmetric dimethylarginine~~dimethyl arginine,atherosclerosis~~, atherosclerosis, lipoprotein (A), and impaired lipid profiles.[2]~~.~~

Hyperhomocysteinemia is~~are~~ associated with increased ADMA ~~concentrationwhich~~concentration, which ~~isconsidered~~is ~~as~~ an endogenous inhibitor of nitric oxide synthase and ~~it~~ synthesized by arginine methylation~~is synthesized bymethylation of arginine~~. It is thought that both ADMA and homocysteine have

adverse vascular effects because they interfere with endothelial, nitric oxide-dependent functions.~~-~~[3]~~.~~ ~~Also,ADMA~~ADMA levels also exacerbate oxidative stress and monocyte adhesion that correlates with the ~~thickness of the~~ carotid media intima thickness (MIT) complex.[4]~~.~~

Over~~More than~~ 15% of children receiving AEDs are at risk of~~for~~ hyperhomocysteinemia. T~~, and t~~his risk may increase by~~with~~ using ~~the use of~~ polytherapy (a combination of 2 AEDs as carbamazepine, ~~valporic~~valproic acid (VP), phenytoin, ~~vigabatrine~~vigabatrin, ~~oxcabazepine~~oxcarbazepine (OXC), topiramate (TPM), lamotrigine (LTG), and clobazam).[5]~~.~~ The available data on the potential effects of new-generation AEDs on hyperhomocysteinemia metabolism, including LTG, OXC, TPM, and ~~levatiracetam~~levetiracetam (LEV), is~~are~~ ~~still~~ relatively ~~small.These~~small. These drugs may be safer than their~~its~~ counterparts of older generations in children with ~~epilepsywho~~epilepsy who are predisposed to developing atherosclerosis early in life, such as those with hereditary diseases of hyperhomocysteinemia metabolism or familial hyperlipidemia.[6]~~.~~

Aim: An e~~E~~valuation of serum Asymmetric Dimethyla~~-A~~rginine (ADMA), homocysteine, lipid profiles, and their correlation to carotid media intima ~~thicknessinepileptic~~thickness in epileptic children receiving valproic~~valporic~~ acid, ~~levetricetam~~levetiracetam, and polytherapy treatment.

Subjects and Methods:

Design:

This case-control~~case control~~ study was carried out on 159 epileptic children that were~~;~~ subdivided into 3 subgroups: (1) ~~53 in each one,~~53 receiving sodium valproate,

(2) 53 receiving levetiracetam, and (3) 53 receiving polytherapy (two or more of ~~valproate,carbamazepine~~valproate, carbamazepine, topiramate, phenytoin, ~~phenobarbital,lamotrigine~~phenobarbital, and lamotrigine) ~~respectively~~ for over~~more than~~ 6 months. There~~y~~ were 100 boys and 59 girls with an age range of (11 months to 8 years) and 50 ~~apparently~~ healthy children.~~,~~ The a~~a~~ge, sex, and socioeconomic ~~standardmatched~~status of this group matched ~~as~~ the control group. The study period~~period of the study~~ was from March 2021 to the first of October 2021. They were recruited from the pediatric~~frompediatriac~~ neurology outpatient clinic, Menoufia University Hospital. After obtaining informed written consent, the~~The~~ Ethics Committee of Menoufia University's Faculty of Medicine approved this study (ID-8/3/2021.PED)~~ after obtaining of an informed written consent~~. This study details the~~Detailed~~ medical histories, ~~and~~ physical ~~examinationsand~~examinations, and the following investigations performed on each patient: ADMA, homocysteine, lipid profiles, and carotid intima-media~~intima media~~ thickness.~~, were performed for each patient.~~

Diagnostic I~~i~~nclusion and E~~e~~xclusion C~~c~~riteria

Inclusion C~~c~~riteria:

Idiopathic epileptic children receiving~~receiving~~ sodium valproate, ~~levetricetam~~levetiracetam, and polytherapy treatment for at least 6 months~~ duration~~. Healthy control has no ~~not any~~ neurological, vascular, or metabolic diseases.

Exclusion C~~c~~riteria:

The exclusion criteria includes the following: sSecondary epilepsy and a short duration of antiepileptic drugsAED less than 6 months.Obesitymonths, obesity and hepatic diseases,. aAny vascular disease that may affect the elasticity or thickness of the vessels (e.g.e.g.,: diabetes mellitus, hypertension, and sickle cell disease), and. aAny metabolic disease that may affect lipid profiles.

Sample Ccollection and Aassay

Sampling: A 4 ml venous blood sample was drawn from each participant under complete aseptic conditions, allowed to clot, and then centrifuged for 15 minutes at 3000 rpm to separate the serum for assessing the biochemical tests (homocysteine, lipid profile, and serum human asymmetrical dimethylargininedimethyl arginine (ADMA)).

Methods: The sSerum homocysteine was measured using (The ARCHITECT i1000SR immunoassay analyzer). The Lipid profiles [total cholesterol, triglycerides, high-density lipoprotein cholesterol (HDL-C),, and low-density lipoprotein cholesterol (LDL-C)] were measured by the Beckman Coulter (Synchron CX 9 ALX).Clinical). Clinical Auto analyzer(analyzer (Beckman Instruments, Fullerton, California). The enzyme-linked immunosorbent assay (ELIZA) was used to determine human asymmetrical dimethylargininedimethyl arginine (ADMA) by using kits provided by the Shanghai Sun Bbright Biological Technology Co., Ltd. Catalogue No. 201-12-1888. Human asymmetrical dimethylargininedimethyl arginine was added to monoclonal antibodies enzyme wells that were had been pre-coatedprecoated with human ADMA monoclonal antibodies, followed by incubation.; then, Hhuman asymmetrical dimethylargininedimethyl arginine antibodies were then labelled with biotin and combined with streptavidin-HRP to form an immune complex.; Fand finally, incubation and washing were repeated to remove the uncombined enzyme. A

and B chromogen solutions were then added. Due to the acid’~~'~~s impact, the liquid becomes blue and eventually yellow. The sample’~~'~~s chroma was ~~shown to be~~ favorably associated with the concentration of the Human Substance Human asymmetrical dimethylarginine~~dimethyl arginine~~. The intra-assay and inter-assay coefficients~~ of~~ variations were 10% and 12%, ~~respectively, ~~corresponding~~which corresponded~~ to the manufacturer’~~'~~s quoted values.

Technique Description of CIMT:

Duplex ultrasound on carotid artery by ESAOTE~~E SAOTE~~ model prestige with transducer 7.5 MHz, Italy.

CIMT measures were obtained w~~W~~hen the patient was laying supine with the neck rotated to the other side of the examination. ~~, CIMT measures were obtained. ~~Using three different ~~angle ~~views of each vessel, CCA images were obtained to determine IMT.~~ ~~[7]~~ .~~There were at least three IMT points measured at each vessel’~~'~~s far and near walls in the thickest part of the ~~vessel.Two~~vessel. Two longitudinal views of the sternocleidomastoid ~~musclewere~~muscle were used to scan the vessel: posterolateral (PL) ~~andanterolateral~~and anterolateral (AL)~~ ~~[8].

Sample S~~s~~ize C~~c~~alculation:

The sample size ~~was ~~relied on~~upon~~ a 95% CI with 80% power, using a 1-way ANOVA (with equal-size groups) and assuming a (two-sided) α of 0.05. Based on a~~the~~ previous study (Khanna et al., 2017)[9], the smallest Mean of ADMA (μM/L) was 1.27 while the largest Mean was 2.10 and SD was 1.38.~~,~~ T~~t~~he number of participants was 53 for each group of epileptic cases.

Statistical Analysis:

IBM SPSS version 20 was used to ~~analyse~~analyze the data (SPSS Inc., Chicago, IL). To examine~~For the purpose of examining~~ the relation between qualitative variables, a chi-square test was applied. For the quantitative data, a comparison between three groups was made~~done~~ using either ANOVA or Kruskal Wallis test (non-parametric test) as appropriate. For correlation between numerical variables, Pearson'~~'~~s correlation coefficient was used. A *p*-value < 0.05 was considered significant.

Results:

There were no significant differences for~~as regard~~ age, sex, hemoglobin, white blood cells, and~~cellsand~~ platelets between patients and control groups, as shown in **(Table 1).**

As for lipid~~Regarding lipid~~ profiles, low-density~~low density~~ lipoprotein (LDL), triglycerides, and cholesterol levels were significantly higher in epileptic children than the control groups~~s~~ (p<0.001), higher in those receiving multidrug followed ~~byvalproate~~by valproate receiver while the levetiracetam group was less ~~affected.While~~affected. In contrast, high-density~~high density~~ lipoprotein (HDL) was lower in those receiving multidrug more ~~thanthose~~than those receiving ~~valproatewith~~valproate with a significant difference compared~~comparing~~ with controls, as shown in **(Table 2)**.

T~~By t~~he analysis of ADMA and~~,~~ ~~homocystiene~~homocysteine ~~levels,both~~levels show ~~markersin patients had~~ a significant increase in both markers inpatients compared to controls (p<0.001). B~~b~~ut within the higher ADMA mean in the multidrug receiver (5.78±0.62) followed by the levetiracetam group (5.56±0.61), the~~while~~ homocysteine levels were significantly higher in multidrug and valproate treated children compared to levetiracetam ones.~~,~~ C~~c~~arotid intima-media~~intima media~~ thickness (CIMT) was also

significantly higher in multidrug and ~~valproatetreated~~valproate treated patients compared to the control group (p<0.001) with no difference in the levetiracetam group (**T~~t~~able 3**).

There was a significant positive correlation ~~betweenserum~~between serum ADMA and triglycerides in the valproate group and with ~~homocystiene~~homocysteine in the multidrug receiver, ~~whilethere~~while there was a highly~~high~~ significant positive correlation between ADMA and HDL in both groups. On the other hand, there was ~~nosignificant~~no significant correlation between ADMA and the~~all~~ parameters in the levetiracetam group. There was a significant positive correlation between ADMA and ~~homocysteine(~~homocysteine (**Table 4).**

Discussion:

To our knowledge, this is one of the most important studies~~study~~ to evaluate the risk of atherosclerosis in children who received old and new AEDs associated with hyperhomocysteinemia~~hyperhemocysteinemia,~~ and elevated ADMA levels~~levelin~~ in our center.

As for the lipid~~Regarding lipid~~ profile (triglyceride, LDL, cholesterol, HDL) study, there was a significant elevation of lipid profiles in patients compared with the controls groups, regardless of~~whatever~~ the type of AEDS received. Comparing~~By comparing~~ patients who received the old drug (group A) with group B showed~~, there was~~ a significant elevation of these markers in group A, while those receiving polytherapy had higher levels compared~~ing~~ to both groups A and B, except for~~exception of~~ HDL that~~HDLthat~~ was not significantly affected in levetiracetam group. Some serum lipids, such as total cholesterol (TC) and LDL-C, promote atherosclerosis, while others~~whereas others~~ act as a strong defense against it (e.g.,

HDL HDL-C). TheIt's the ratio between the cholesterol fractions (TC/HDL and LDL/HDL) isthat are a better indicator for developing the development of atherosclerosis in patients receiving long--term anticonvulsants.[10].

The aAntiepileptic medications' effects on the lipid profiles were controversial in previousthepreviousstudies.Somestudies. Some of these studies illustrated an elevation of triglycerides and HDL levels.[11, 12, 13];. Aalso–, there waswere an elevation of LDL values.[14,15]b. But, Eiris et al.[11] reported a decreasedecreased in LDL levels in -patients treated with AEDS.

The possible explanation forto decreased serum lipids with valproate is valproate''s enzyme inhibitory effect. GlucouronidationGlucuronidation is the major route of valproate biotransformation.Valproatebiotransformation. Valproate or its metabolites may inhibit the glucouronidazeglucuronidase enzyme, resulting in reduced triglyceride, LDL, and HDL production.[11].

Also,HorieHorie and Suga[16] also observed that valproate treatment increased hepatic peroxisomal oxidation, reducingresulting in a reduction in LDL-c and apolipoprotein B. Valproate-induced weight gain may result in insulin resistance, resulting in dyslipidemia and hyperinsulinemia.[17].

Akosy et al.[18] observed that the serum lipid profile and thyroid function tests did not affect long-term LEV administrationget affected after long-term administration of LEV. As a result, it appears that LEV outperforms valproate in terms of benefits.Howeverbenefits. However, **Kim et al.**[19] showed a significant increase in LDL-C levels in LEV-treated patients.Nopatients. But no effect of LEV was seen onvitaminon vitamin B12, triglyceride, total cholesterol, or HDL-C levels. There was a highlyhigh significant increase in homocysteine levels in patients compared to

~~control;this~~those in the control group. These higher ~~levelwas~~levels were reported in the multidrug group followed by the valproic acid group.[(20)]~~.~~

The mechanism of valproate that induces~~inducing~~ hyperhomocysteinemia is not fully understood.[(21)] ~~.~~Moreover, the results of the possible effects of new-generation AEDs, such as OXC, lamotrigine, topiramate, and ~~levatiracetam~~levetiracetam, on the metabolism of homocysteine are limited~~remains limited.~~[(22)]~~.~~ **Belcastro et al.~~al~~**[(~~22~~32)]~~,~~ demonstrated that ~~the~~ newer AEDs as TPM and OXC may lead to hyperhomocysteinemia, while AEDs as LEV and LTG had no ~~did not have any~~ effect on homocysteine level.

The relationship between the use~~usage~~ of ~~the~~ new AEDs and homocysteine levels was studied in Korean patients with newly diagnosed epilepsy and treated with OXC, ~~levatiracetam~~levetiracetam, or TPM as monotherapy.~~,~~ **Kim et al.**[(19)] observed a statistically significant~~statistical significant~~ elevation in ~~homocysteineconcentration~~homocysteine concentration throughout each drug'~~'~~s therapy, but these changes are within the physiological concentrations range.

Gorgone et al.~~al~~[(~~24~~23)] reported that 30% of patients ~~found~~ with brain atrophy were ~~and that was~~ associated with the use ~~of a combination~~ of different AEDs and an elevated homocysteine concentration. So~~So,~~ there was a correlation between homocysteine-induced neuronal injury, oxidative stress, and excitotoxicity.

In another research by **Ono et al.**[(2~~5~~4)], they found an increased risk of hyperhomocysteinemia in ~~those~~ patients ~~who were takingprolonged~~taking prolonged, multiple AED therapies~~y~~ over~~more than~~ 7 years (>7 years). Also, **Vilaseca et al.**[(2~~6~~5)] found elevated total homocysteine levels~~level~~ in children with epilepsy using~~who used~~ AEDs for an extended period~~period of time~~. These prospective studies were

conducted to ~~assessthat~~assess the ~~timeneeded~~time needed until~~till~~ reaching the hyperhomocysteinemia cutoff value ~~forby~~for using certain AEDs that induce hyperhomocysteinemia. So, long-term morbidity associated with AEDs can be prevented by switching these drugs before reaching the critical cutoff value for hyperhomocysteinemia.

Perhaps~~It is possible that~~ AED~~-~~ gene interactions have a role in the development of hyperhomocysteinemia; patients getting CBZ or PHT have~~had~~ higher ~~levels of~~ homocysteine levels if they were homozygous TT genotype, while those~~whereas those~~ taking valproic acid had lower levels.[26] ~~.~~In contrast, **Vurucuet al**[27] did not confirm the ~~correlationbetween~~correlation between the ~~genotypesof~~genotypes of 677 T variants of hyperhomocysteinemia and the methylenetetrahydrofolatereductase gene ~~polymorphismin~~polymorphism in patients with epilepsy and treated with valproate monotherapy and CBZ.

ADMA levels in the patient groups were significantly higher than the control group~~,~~ but remained~~remains~~ within normal levels (< 15 µmol/l), with higher levels in the multidrug group than the other groups~~group~~ and ~~showed~~ a significant positive correlation with ~~homocysteine.**Khanna**~~homocysteine. **Khanna et al.~~al~~**[9] found an elevation of~~elevated~~ both ADMA and homocysteine levels in children receiving valproate and OXC after six months of therapy. Similarly~~Similarity~~, **Oz et al.~~al~~**[28] illustrated the effect of valproate and OXC therapy on the ADMA levels in the Indian population. Hyperhomocysteinemia leads to increased ADMA production, which lowers the nitric oxide levels increasing the risk of atherosclerosis.[29,30] ~~.~~ A significant elevation of ADMA levels was reported in children receiving valproate[2], **Sniezawska et al.~~al~~**[31] observed a significant link between asymmetric dimethylarginine (ADMA) levels and hyperhomocysteine in children with

~~epilepsy.On~~epilepsy. Yet ~~the other hand,~~ another study reported a significant increase in ~~both~~ ADMA and homocysteine levels in children on OXC ~~therapywith~~therapy with no significant correlation between ADMA and homocysteine.[32]~~.~~

Carotid intima-media~~intima media~~ thickness (CIMT) was significantly higher in patients receiving ~~received~~ multidrug and valproate compared to the control group, with no difference in the levetiracetam group. **Ksoo et al.~~al~~** [(33)] illustrated a significant increase in CIMT values in children receiving phenytoin and carbamazepine Carbamazepine after 3 months of therapy. Recent studies showed that patients with epilepsy who received AEDs might~~may~~ exhibit an ~~increasedrisk~~increased risk of ~~the~~ myocardial infarction, stroke, and cardiovascular death that may be triggered by affecting ADMA and homocysteine concentration and~~as well as the~~ serum lipid levels.[34, 35, 36]~~.~~

Limitations of the study: Because patients were not screened~~had not screened~~ genetically for the CBS ~~genesand~~genes and MTFR, both ~~of which are~~ known to have a role in homocysteine ~~metabolism.So~~metabolism, we recommend the genetic workup of homocysteine and ADMA for early detection of the risk factors of vascular disease in children receiving anti-epileptic drugs for a long duration.

~~**Conclusion:**Long~~**Conclusion:** Long-term~~-term~~ use of antiepileptic drugs, especially old generation polytherapy, have risks of elevated~~ion~~ ~~of~~ lipid~~s~~ profiles, homocysteine~~homocystien~~ levels, ADMA, and increased~~increase~~ carotid intima thickness compared to the minimal effect of new antiepileptic generation.~~, and~~ R~~r~~outine follow-up~~follow up~~ of these markers is recommended with ~~life style~~lifestyle modification to avoid cerebrovascular events as much as possible.

~~**Acknowledgements:**~~~~The~~**Acknowledgments:** The authors would like to express their gratitude to the participants who participated~~took part~~ in the study and ~~to~~ the data collecting team.

References:

[1] French J. Treatment with antiepileptic drugs, new and old. Continuum: Lifelong Learning in Neurology. 2007; 13:71-90.

[2] Ozdemir O, Yakut A, Dinleyici EC, Aydogdu SD, Yarar C, Colak O. Serum asymmetric dimethyl arginine (ADMA), homocysteine, vitamin B12, folate levels, and lipid profiles in epileptic children treated with valporic acid. European Journal of Pediatrics 2011; 170: 873–7.

[3] Vallance P, Leiper J. Cardiovascular biology of the asymmetric dimethyl arginine: dimethyl arginine dimethyl amino-hydrolase pathway. Arterioscler Thromb Vasc Biol. 2004; 24:1023–1030.

[4] Ayer JG, et al. HDL-cholesterol, blood pressure, and asymmetric dimethyl arginine are significantly associated with arterial wall thickness in children. Arterioscler ThrombVascBiol.2009; 29(6):943-949.

[5] Huemer M, et al. Hyperhomocysteinemia in children treated with anti-epileptic drugs is normalized by folic acid supplementation. .
Epilepsia. 2005; 46(10):1677-1.

[6] Jakubus T, Michalska-Jakubus M, Lukawski K, Janowska A, Czuczwar SJ. Atherosclerotic risk among children taking antiepilepticdrugs. Pharmacological Reports 2009; 61:411–23.

[7] Seçil M, Altay C, Gülcü A, Ceçe H, Göktay AY, Dicle O, Automated measurement of intima-media thickness of carotid arteries in ultrasonography by computer software. Diagn Interv Radiol. 2005; 11:105–8.

[8] Simon A, Gariepy J, Moyse D, Levenson J. Differential effects of nifedipine and co-amilozide on the progression of early carotid wall changes. Circulation. 2001 ; 103:2949–54.

[9] Khanna N, Verma SL, Maurya PK, Tiwari V, Kulshreshtha D, Effect of Valproate and Oxcarbazepine therapy on asymmetric dimethyl arginine (ADMA) and Homocysteine levels in newly diagnosed epileptic children. Journal of Advanced Medical and Dental Sciences Research, 2017; 5(7):57-63. DOI: 10.21276/jamdsr.2017.5.7.15.

[10] Zeitlhofer S, Doppelbauer A, Tribi G, Leitha T, Deecke L. Changes of serum lipid pattern during long term anticonvulsant treatment. Clin Invest; 1993; 71: 574-8.

[11] Eirís J, INovo-Rodríguez M, MDel Río PMeseguer, M. CDel Río MCastro-Gago. The effects on lipid and apolipoprotein serum levels of long-term

carbamazepine, valproic acid and phenobarbital therapy in children with epilepsy, Epilepsy Research 2000; 41 (1): 1-7.

[12] Müjgan A.F.M, Orhan F, Orem A, Yildirmis S, Gedik Y, Effect of Antiepileptic Drugs on Plasma Lipoprotein (a) and Other Lipid Levels in Childhood Journal of child neurology 2001; 16: (5).

[13] Nikolaos T, et al. The effect of long-term antiepileptic treatment on serum cholesterol (TC, HDL, LDL) and triglyceride levels in adult epileptic patients on monotherapy. Med Sci Monit. 2004; 10 (4):MT50-2.

[14] De Juan J, Crespo M, Braga S. Efectos del fenobarbital, acido valproico y carbamacepina sobre lipidos y lipoproteinas sericas en poblacion infanto-juvenil. An Esp Pediatr; 1996; 44: 133-138.

[15] Sozuer DT, Atakli D, Dogu O, Baybas S, Arpaci B, Serum lipids in epileptic children treated with carbamazepine and valproate. Eur J Pediatr; 1997; 156: 565-567.

[16] Horie A, Suga T, Enhacement of peroxisomal beta oxidation in the liver of rats and mice treated with valproic acid. Biochem Pharmacol, 1985; 34, 1357–1362.

[17] Morrell MJ, et al. Higher androgens and weight gain with valproate compared with lamotrigine for epilepsy. Epilepsy Res 2003; 54, 189–199.

[18] Aksoy D, Solmaz V, Çevik B, Pekdaş E, Kurt S. Serum Lipids and Thyroid Functions in Young Epileptic Patients Undergoing Monotherapy with Valproate or Levetiracetam, Eur J Gen Med 2015; 12(1):59-63.

[19] Kim DW, Lee SY, Shon YM, Kim JH. Effects of new antiepileptic drugs on circulatory markers for vascular risk in patients with newly diagnosed epilepsy. Epilepsia 2013; 54(10):e146-9.

[20] Siniscalchi A, et al. Increase in plasma homocysteine levels induced by drug treatments in neurologic patients. Pharmacological Research 2005; 52:367–75.

[21] Jakubus T, Michalska-Jakubus M, Lukawski K, Janowska A, Czuczwar SJ. Atherosclerotic risk among children taking antiepileptic drugs. Pharmacological Reports 2009; 61:411–23.

[22] Belcastro V, et al. Hyperhomocysteinemia in epileptic patients on new antiepileptic drugs. Epilepsia 2010; 51:274–9.

[23] Gorgone G, et al. Hyperhomocysteinemia in patients with epilepsy: does it play a role in the pathogenesis of brain atrophy? A preliminary report. Epilepsia. 2009; 50(1): 3-36.

[24] Ono H, et al. Plasma total homocysteine concentrations in epileptic patients taking anticonvulsants. Metabolism Clinical and Experimental 1997; 46:959–62.

[25] Vilaseca MA, et al. Anti-epileptic drug treatment in children: hyperhomocysteinaemia, B-vitamins and the 677C ! T mutation of the methylenetetrahydrofolate reductase gene. European Journal of Paediatric Neurology 2000; 4:269–77.

[26] Ni G, et al. Effects of anti-epileptic drug monotherapy on one-carbon metabolism and DNA methylation in patients with epilepsy. PLoS One. 2015;10 (4):e0125656.

[27] Vurucu S, et al. Evaluation of the relationship between C677 T variants of methylenetetrahydrofolate reductase gene and hyperhomocysteinemia in children receiving anti-epileptic drug therapy.

Prog Neuropsychopharmacol Biol Psychiatry. 2008; 32(3):844-848.

[28] Öz O, Gökçil Z, Bek S, Çakır E, Odabaşı Z. Is asymmetric dimethyl arginine responsible for the vascular events in patients under antiepileptic drug treatment? Epilepsy research. 2009; Nov 30; 87(1):54-8.

[29] Lentz SR, Rodionov RN, Dayal S. Hyperhomocysteinemia endothelial dysfunction, and cardiovascular risk: the potential role of ADMA. Atherosclerosis supplements. 2003; Dec 31; 4(4):61-5.

[30] Dayal S, et al. Tissue-specific down regulation of dimethyl arginine dimethyl amino-hydrolase in hyperhomocysteinemia. American Journal of Physiology-Heart and Circulatory Physiology. 2008; Aug 1; 295(2):H816-25.

[31] Sniezawska A, et al. MTHFR, MTR, and MTHFD1 gene polymorphisms compared to homocysteine and asymmetric dimethylarginine concentrations and their metabolites in epileptic patients treated with antiepileptic drugs. Seizure; 2011; 20:533–540. DOI: 10.1016/j.seizure.2011.04.001.:

[32] Emeksiz HC, et al. Assessment of atherosclerosis risk due to the homocysteine–asymmetric dimethyl arginine–nitric oxide cascade in children taking antiepileptic drugs. Seizure. 2013 Mar 31; 22(2):124-7.

[33] Ksoo R, Sharma R, R. K. Kaushal, Jhobta A. The effects on carotid artery intima-media wall thickness and development of atherosclerosis in children on anti-epileptic drug monotherapy. Int J Contemp Pediatr. 2017 Jul; 4(4):1369-1373.

DOI: http://dx.doi.org/10.18203/2349-3291.ijcp20172668.

[34] Olesen JB, et al. Effects of epilepsy and selected antiepileptic drugs on risk of myocardial infarction, stroke, and death in patients with or without previous stroke: a nationwide cohort study. Pharmacoepidemiology and Drug Safety; 2011, 20: 964–971. DOI: 10.1002pds.2186.

[35] Renoux C, Dell'Aniello S, Saarela O, Filion KB, Boivin JF. Antiepileptic drugs and the risk of ischemic stroke and myocardial infarction: a population-based cohort study. BMJ Open; 2015; 5:e008365. DOI: 10.1136/bmjopen-2015-008365.

[36] Vyas MV, et al. Antiepileptic drug use for treatment of epilepsy and dyslipidemia: systematic review. Epilepsy Research; 2015; 113:44–67. DOI: 10.1016/j.eplepsyres.2015.03.002.

Figure Legend: Correlation between ADMA and other parameters among each group
